# Sustainable Elimination (Zero Cases) of Sleeping Sickness: How Far Are We from Achieving This Goal?

**DOI:** 10.3390/pathogens8030135

**Published:** 2019-08-29

**Authors:** Pearl Ihuoma Akazue, Godwin U. Ebiloma, Olumide Ajibola, Clement Isaac, Kenechukwu Onyekwelu, Charles O. Ezeh, Anthonius Anayochukwu Eze

**Affiliations:** 1 Department of Biochemistry, Faculty of Life Sciences, University of Benin, Benin City 300283, Nigeria; 2 Institute of Infection, Immunity and Inflammation, College of Medical, Veterinary and Life Sciences, University of Glasgow, Glasgow G12 8TA, UK; 3 Medical Research Council Unit The Gambia at London School of Hygiene and Tropical Medicine, Banjul PO Box 273, The Gambia; 4 Department of Zoology, Faculty of Life Sciences, Ambrose Alli University, Ekpoma 310101, Nigeria; 5 Department of Medical Biochemistry, Faculty of Basic Medical Sciences, University of Nigeria, Enugu Campus, Enugu 410001, Nigeria

**Keywords:** Human African Trypanosomiasis, elimination, *T. b. gambiense*, *T. b. rhodesiense*

## Abstract

The recent massive reduction in the numbers of fresh Human African Trypanosomiasis (HAT) infection has presented an opportunity for the global elimination of this disease. To prevent a possible resurgence, as was the case after the reduced transmission of the 1960s, surveillance needs to be sustained and the necessary tools for detection and treatment of cases need to be made available at the points of care. In this review, we examine the available resources and make recommendations for improvement to ensure the sustenance of the already achieved gains to keep the trend moving towards elimination.

## 1. Introduction

*Trypanosoma brucei gambiense* (*T. b. gambiense*) and *Trypanosoma brucei rhodesiense* (*T. b. rhodesiense*) are the causative agents for the Human African trypanosomiasis (HAT), and together with *Trypanosoma brucei brucei* (*T. b. brucei*) are the sub-species of *Trypanosoma brucei*. *T. b. brucei*, *Trypanosoma congolense* (*T. congolense*) and *Trypanosoma vivax* (*T. vivax*) cause nagana in cattle. *Trypanosoma evansi* (*T. evansi*) is responsible *surra* in camels and also cause disease in a wide range of domestic and wild animals such as camelids, equines, cattle, buffaloes, sheep, goats, pigs, dogs, deer, gazelles, elephants, etc [1], *Trypanosoma equiperdum* (*T. equiperdum*) is responsible for dourine of horses [2], while *Trypanosoma simae* (*T. simae*) and *Trypanosoma suis* (*T. suis*) are the causal organisms for trypanosomiasis in pigs. African trypanosomiasis occurring in animals, livestock and wild animals, is known as Animal African Trypanosomiasis (AAT).

Sleeping sickness propagated by *T. b. rhodesiense* progresses swiftly while the *T. b. gambiense* sleeping sickness develops more slowly. Symptoms usually includes fever, headache, muscle and joint aches, enlarged lymph nodes, etc., (https://www.cdc.gov/parasites/sleepingsickness/disease.html). Parasite invasion of the central nervous system occurs in a few weeks (for *T. b. rhodesiense* infection) or in a few years (for *T. b. gambiense* infection), leading ultimately to coma and death if untreated. HAT is a Neglected Tropical Disease (NTD) that was an epidemic a few decades ago, but is now becoming a rare disease (https://www.who.int/trypanosomiasis_african/news/progress-on-eliminating-hat-as-public-health-problem/en/). We review the activities in the run-up to this achievement, the resources available and what is needed to sustain this level of control in the campaign for the elimination of HAT.

## 2. Human Infectivity

Most of the African trypanosomes, except for *T. b. gambiense* and *T. b. rhodesiense*, are sensitive to apolipoprotein-L1-mediated lysis by the human serum [3]. Evasion of this lysis by *T. b. rhodesiense* in human serum is by expressing the serum resistance-associated (SRA) protein, which binds apolipoprotein-L1 in the endosomal-lysosomal system and prevents pore formation [4]. However, resistance to lysis by *T. b. gambiense* is a multifactorial process involving the uptake of reduced trypanolytic factor (TLF1) by an alternative form of *T. b. gambiense* haptoglobin-haemoglobin receptor (TbHpHbR), *T*. *b*. *gambiense*-specific glycoprotein (TgsGP) mediated endosomal membrane stiffening and a rise in the lysosomal cysteine protease activity [5,6].

## 3. Parasite Life Cycle

*T. brucei* enters the mammal to start its lifecycle during a bloodmeal by an infected tsetse fly, which injects the metacyclic trypomastigote forms of the parasite in its saliva into the skin [7]. This injection is accompanied by the formation of a skin lesion or chancre at the position of the tsetse bite. The chancre which appears as early as the fifth day post-infection varies in diameter from a few millimeters to several centimeters [8] and is denoted by a severe host inflammatory reaction usually associated with *T. b. rhodesiense* infection and usually resolves within three weeks of infection [9]. The chancre marks the invasion of the lymphatic organs by the trypanosomes. After deposition in the skin, metacyclics develop into the long slender bloodstream forms that populate the blood and tissue fluids of mammals [10]. The long slender bloodstream forms can survive the immune attack of the host because they express variant surface glycoproteins (VSG) on their cell surface [11,12,13]. At maximum parasitemia, the bloodstream form trypanosomes differentiate into the short stumpy form, the form best suited for survival in the tsetse fly [14]. The adaptation in the short stumpy form includes a change from the parasite’s metabolic requirement for glucose to a dependence on proline which is abundant in the tsetse fly midgut [15]. In the midgut of the tsetse fly, the short stumpy forms develop into the procyclic forms with a change in the surface coat expression from VSG to a less dense EP (marked by an internal repeat of glutamic acid E and proline P) and GPEET (marked by an internal repeat of glycine G, proline P, glutamic acid E, and Threonine T) procyclin [16]. Following multiplication in the midgut or proventriculus, procyclic trypanosomes migrate toward the salivary glands, undergoing an asymmetric cell division to produce a long epimastigote form and a short epimastigote form [17]. Only the short forms are thought to be destined for attachment and proliferation in the fly salivary gland, where EP procyclin mRNA continues to be expressed although procyclin protein is not detectable on most parasites. This repression is due to sequences present in the procyclin coding region [18]. Epimastigotes are thought to be the stage at which sexual exchange occurs between trypanosome lines, with hybrids being detectable in the salivary gland after parasite lines tagged with distinct fluorescent reporters were transmitted through tsetse flies [19]. The epimastigotes develop into the metacyclics and reacquire the VSG coat [20]. After detaching from the salivary gland wall, these metacyclics are infectious to mammals and can be transmitted during a tsetse blood meal.

## 4. Transmission of HAT

It was observed that though tsetse fly of all species can transmit HAT, the tsetse fly belts are wider in Sub-Saharan Africa (SSA) than the HAT distribution [21], resulting in areas with the vector but no disease. Also, tsetse flies infected with *T. b. gambiense* have been found to produce midgut infections that fail to develop into mature salivary gland infections [22,23]. Similarly, only 30% of six field isolates of *T. b. gambiense* were able to follow through with the life cycle to the salivary gland stage [24] in *Glossina palpalis gambiensis*. Conversely, 27 serum-resistant *T. brucei* strains including one strain isolated from the tsetse fly, nine strains from cattle (all 10 strains confirmed to be distinct from *T. b. gambiense*) and 17 *T. b. rhodesiense* all were able to produce the metacyclic salivary gland trypanosomes in tsetse fly 28-days post-infection [25]. These reports suggest that tsetse fly efficiently transmits *T. b. rhodesiense* but not *T. b. gambiense*. Congenital infection with HAT results from mother to child transmission and is specifically the diagnosis of HAT in a newborn of an infected mother in the first five days of life [26,27]. Cases of vertical transmission of HAT reported are mostly due to *T. b. gambiense* infection [26,28,29], likely because of the chronic nature of its infection. An interesting case of a twin birth from a mother suffering from first stage HAT infection challenges our definition of a congenital infection. One of the twins was diagnosed with HAT at birth while the second twin was diagnosed six weeks later [30]. Similarly, a 19-month-old son born to a white Portuguese lady (living in the United States) who had never been to Africa but had a Brazilian partner who was in Angola on a military mission, was diagnosed with stage 2 gHAT infection. The lady and her partner were also diagnosed with the same infection and treated [31]. These reports suggest that the diagnosis of congenital infection may not always be possible in the first five days of life, and the Portuguese lady’s infection could only have been by sexual transmission. Vertical transmission seems to be a rare event, but this rarity may be due to the underestimation of this means of transmission, resulting from missed opportunities for diagnosis because HAT screenings are usually done in villages, and not in hospitals or well-equipped laboratories [32]. Also, routine tests for HAT in pregnant women are not being carried out to diagnose and treat infections in pregnant women to reduce vertical transmission. 

## 5. Epidemiology

The most recent trypanosomiasis epidemic occurred in 1970, with most of the cases occurring in Angola, Congo, Southern Sudan, and the West Nile area of Uganda, and lasted till 1990 when eflornithine was registered for use in place of melarsoprol for treatment of stage 2 *T. b. gambiense* infection [33]. During the period of the epidemic, sleeping sickness replaced HIV/AIDS as the greatest cause of death in many communities in Angola, South Sudan, and the Democratic Republic of Congo (DRC) where prevalence of up to 50% was reported [34]. World Health Organization (WHO) established public-private cooperations with Aventis Pharma (now Sanofi) and Bayer HealthCare in 2000 and 2001 respectively, and renewed in 2006, 2011, and 2016, which enhanced the creation of a WHO surveillance team, supported endemic countries in their control activities and provided free drugs for treatment of patients [34]. As a result, the total number of new HAT infections reported per year in Africa was reduced from 37,991 in 1998 to 1,446 in 2017, with the actual number of cases estimated at below 10,000 [34]. These numbers are however very likely lower than the true actual number of cases, since undiagnosed deaths due to sleeping sickness were not considered [35]. 

The WHO in 2012 set the goal of reducing the impact of sleeping sickness to the level where it ceases to be a public health problem by 2020 in its NTD roadmap [36]. One of the indicators to monitor the achievement of this goal was the reduction of the annual number of new cases of HAT to <2000 by the year 2020 [37]. This goal has already been achieved, thus paving the way for the 2030 goal of total elimination of transmission of the *gambiense* HAT (gHAT) to achieve zero cases [38]. 

## 6. Diagnostic Tools

Card-agglutination trypanosomiasis test (CATT) is a serological test used for the preliminary screening for gHAT. It is a cheap, fast test that can be run on fresh or dried blood, plasma or serum [39]. Diagnosis of gHAT infection is subsequently confirmed by microscopic identification of trypanosomes in blood, lymph nodes or cerebrospinal fluid. CATT tests for *T. b. gambiense* variable surface antigen LiTat 1.3 [40]. A reduction in disease burden, as is the case with the HAT now, causes a depreciation in the positive predictive value of the available diagnostic tests for HAT such as the card agglutination test for trypanosomes, CATT [41]. In addition, serologic tests are not useful for following the course of treatment because they give false-positive results, after cure, with antibodies from the previous exposure. New tests with enhanced sensitivity for HAT detection, therefore, needs to be developed. Antigen evaluation for new serologic tests found the invariant surface glycoproteins, ISG64 and ISG65, to be quite reactive to sera from *gambiense* patients while the SRA displayed a strong reactivity to sera from *rhodesiense* patients. VSG LiTat 1.3 and VSG LiTat 1.5 were found to be the among the most reactive antigens against both the *gambiense* and the *rhodesiense* sera samples [42]. However, none of the antigens tested was able to react with all positive sera for either *gambiense* or *rhodesiense*, suggesting that at least two antigens need to be combined to develop a test with the sensitivity for effective disease control. Molecular detection of trypanosome cell-free DNA [43] by loop-mediated isothermal amplification (LAMP) technique is highly specific and sensitive, requires minimal equipment since DNA is amplified at a uniform temperature, and can easily be performed by staff with minimal molecular biology experience [44]. The Foundation of Innovative New Diagnostics (FIND) developed a LAMP kit for HAT, in association with Eiken Chemical Company of Japan in 2011. The reaction tubes arrive containing the dried HAT LAMP reagents and can be stored at room temperature, with the addition of the test sample (fresh or dried blood), the tubes are heated at a constant temperature and the result visualized under LED light [44]. 

## 7. Vaccine Prospect

Despite considerable research, not a single vaccine has so far been developed against either the HAT or the AAT. Trypanosomes can stay one step ahead of the host immune response and avoid complete destruction by antigenic variations, which it achieves through VSG switching [11,12,13]. Other not-so-variable surface proteins were studied, and some were reported as promising vaccine candidates. For instance, recombinant p15, an intracellular protein of *T. b. brucei* was found to provide 100% protection, while the native p15 protein provided 87.5% protection from *T. b. brucei* when used to vaccinate mice [45]. However, adenovirus particles lacking p15 (negative control) also provided a similar level of protection as p15, suggesting that this protection was not mediated by immune memory cells. It was suggested that such nonspecific immunity conferred by recombinant proteins might be as a result of contamination with E. coli proteins [46]. Trypanosomes are efficient in circumventing destruction by the host’s humoral immunity by eliminating the B-cell memory and sheltering the conserved epitope in preparation for persistent infection [47]. This preliminary elimination of B-cell memory during infection might be a factor in the failure to develop an efficacious vaccine. Hence, immunization with the immunodominant VSG failed to produce a comprehensive defense [47]. A successful attempt to produce an effective vaccine will require dealing with the ability of the trypanosomes to suppress the host immune response at the initial stage of the infection just after introduction into the skin by infected tsetse flies [48]. However, since there has been such significant progress made towards the elimination of HAT without any vaccine, it is pertinent to consider if vaccines are indispensable to the total elimination of this disease. Also, the lessons learnt from combating the HAT could be applied to other diseases, such as malaria, where the effort to develop vaccines is stalling.

## 8. Chemotherapy

Control of the HAT leans heavily on chemotherapy in the absence of a vaccine. The clinical progression of the 2 forms of HAT involves two stages, namely stage 1 (early-stage, haemolymphatic) and stage 2 (late-stage, meningoencephalitic), and each stage responds to a few of the available trypanocides. Progression to stage 2 of rHAT occurs within a couple of weeks while gHAT advances more slowly to the late stage, usually within months or years post-infection [49]. Approved drugs for the treatment of HAT include pentamidine and suramin for early-stage gHAT while suramin is the only drug for stage 1 rHAT. Melarsoprol, eflornithine, and nifurtimox-eflornithine combination therapy (NECT) are the approved treatments for stage 2 gHAT, while late-stage rHAT currently responds to treatment with only melarsoprol [50]. 

Pentamidine is taken up into the trypanosome cell through the P2 adenosine transporter [51] or the high-affinity pentamidine transporter (HAPT1 or trypanosome aquaporin 2) and kills the parasite by disruption of the mitochondrial genome [52], loss of both the kinetoplast and the mitochondrial membrane potential [53]. Another report found that pentamidine binds to and inhibits trypanosome aquaporin 2 [54]. Melarsoprol seems to react with the trypanothione dithiol to form an adduct known as Mel T which is a mildly strong competitive inhibitor of the parasite’s antioxidant enzyme, trypanothione reductase [55]. Melarsoprol was also found to inhibit mitosis [53]. Eflornithine exerts its trypanostatic effect by inhibiting ornithine decarboxylase in trypanosomes, causing a reduction in polyamine biosynthesis [56] which leads to a fall in the biosynthesis of the trypanosome specific redox-active metabolite, trypanothione. Uptake of Suramin in trypanosomes through an endocytic pathway involves the expression of a bloodstream form-specific invariant surface glycoprotein ISG75 [57], and a variant surface glycoprotein [58]. The mechanism of action of suramin involves the inhibition of cytokinesis [53] and is amplified by the uptake of ornithine through two amino acid transporters and its decarboxylation, by ornithine decarboxylase, which is inhibited by eflornithine, hence antagonizing the action of suramin [59]. Nifurtimox is a prodrug activated by an NADH-dependent, mitochondrially localized, type I nitroreductase (NTR) to exert its cytotoxic effect [60], which includes the disruption of the parasite’s mitochondrial membrane potential [53].

These treatments each require intravenous or intramuscular administration, except for nifurtimox which can be taken orally as a component of NECT (while the eflornithine component is still delivered intravenously). These drugs are considered suboptimal due to high production cost, toxicity, poor oral bioavailability, difficulty in drug administration, long treatment regime and low-efficacy [61]. Melarsoprol, for instance, is so toxic that up to one of every 20 late-stage HAT patients that receive melarsoprol treatment die of a reactive encephalopathy [61]. The nifurtimox-eflornithine combination therapy (NECT) was brought in because of its lower toxicity and treatment period compared to melarsoprol or eflornithine alone, but NECT still required seven days of intravenous administration [62], making it unsuitable for use in resource-poor settings where HAT is endemic.

There is also the problem of drug resistance which greatly reduces the efficacy of the current trypanocides. Pentamidine, and melarsoprol, resistance is developed as a result of a mutation or loss of the trypanosome aquaporin 2 [52,63]. Eflornithine resistance arises due to the loss of TbAAT6 [64], the gene encoding a low-affinity transporter for uncharged amino acids [65]. Resistance to nifurtimox is a consequence of the down-regulation or reduced activity of NTR [60]. 

New chemotherapeutic options recently added to the arsenal of HAT trypanocides include fexnidazole, an orally active nitroimidazole, and the benzoxaborole, SCYX-7158. These will make up for the shortcomings of the currently licensed trypanocides. Fexinidazole is potentially efficacious against both *T. b. gambiense* and *T. b. rhodesiense* HAT and active against the two stages of the disease [61]. However, fexinidazole resistance was easily selected for experimentally, thus making a case for fexinidazole to be used in combination with an unrelated trypanocide [66]. Approval for the marketing of fexinidazole in the DRC as a once-daily treatment for 10 days against *T.b. gambiense* HAT has been granted, making fexinidazole the first new chemical substance to be registered by DND*i* (https://www.dndi.org/2019/media-centre/press-releases/fexinidazole-sleeping-sickness-approved-democratic-republic-congo/). As a safe oral trypanocide, fexinidazole would simplify HAT treatment, and if found to be safe for pregnant and nursing mothers, would help combat infections due to vertical (maternal) transmission of HAT. Similarly, SCYX-7158 (acoziborole) is a single-dose oral tablet for both disease stages of HAT [67], which is expected to be suitable for administration at home (https://www.dndi.org/diseases-projects/portfolio/acoziborole/). A couple of other compounds, including SCYX1330682, SCYX-1608210, and SCYX-2035811 have been kept on hold and will only be developed in the future if the need arises [50]. 

## 9. Prospects for New Drugs

Due to the ever-increasing incidence of drug resistance and the unsatisfactory nature of current antitrypanosomal drugs, there is a great need for new compounds to be developed into drugs [68,69]. There have been rising interests in the use of natural products as starting materials since they form the basis of traditional medications and provide a rich plethora of chemicals that hold potential as new drug leads [70]. Also, they tend to be structurally diverse (providing a vast array of compounds most probably with distinct resistance profiles from the existing drugs) and have low cytotoxicity, high selectivity, and good efficacy [71]. Natural products are secondary metabolites that have been isolated from natural sources, notably plants and micro-organisms. Some earlier reviews have identified natural compounds with antitrypanosomal activity [72,73]. This section focuses therefore on natural products that have been tested against different *Trypanosoma* species in the last decade with promising trypanocidal activities (Table 1). From the table, Abruquinones D, A, and L, isoflavonoids from the plant *Abrus precatorius,* have the lowest IC_50_ values (tested against *Trypanosoma brucei rhodesiense,* the causative agent of rHAT) with good selectivity (selectivity index >300). However, it is worthy to note that differences between different laboratories in IC_50_ values of standard drugs used as controls make it rather challenging to make a direct comparison between compounds in order to determine the most active compound. Though some compounds have antitrypanosomal activity worth exploring further, their poor selectivity does not make them suitable candidates for further studies (for example, Perovskone B and Cynaropicrin). For most of these compounds, there is a need for a mode of action studies to be carried out as this would give insight on how the compounds act as well as biochemical processes that could be targeted in the parasites. While natural products might hold some promise for new treatments, the process of isolation and characterization of bioactive entities is laborious, and sometimes bioactivity is compromised during the isolation process. Also, if the starting materials used for the isolation is insufficient, minimal quantities of bioactive compounds are obtained with limited compounds for follow-up studies. Difficulties are often encountered synthesizing natural products due to the complexity of their structures and in some cases, low solubility. Despite these challenges, natural products remain very vital starting points in the quest for new treatments.

## 10. Vector Control

Tsetse flies belong to the genus Glossina which includes three subgenera (namely, subgenus *Nemorhina*, also known as the *Palpalis* group, subgenus *Glossina* sensu stricto, or the *Morsitans* group, and subgenus *Austenina* also called the *Fusca* group) that together contain 31 species and sub-species, all of which can potentially transmit trypanosomes, but the most important epidemiologically are the *Morsitans* and *Palpalis* groups, found mostly in natural savannahs and riverine forest vegetation respectively [88,89,90]. gHAT is almost totally transmitted by either *Glossina fuscipes* or *Glossina palpalis* subspecies while rHAT can be transmitted by any of *G. fuscipes* SL, *G. swynnertoni*, *G. morsitans morsitans* and *G. pallidipes*, but AAT transmission seems to be geographically dependent, with the *Mortisans* transmitting in East and South Africa while *Palpalis* group dominate West Africa [90]. 

Tsetse control can be carried out to achieve an integrated control or total eradication of the vector [91]. Integrated control of tsetse fly is carried out by livestock farmers using either the insecticide-treated cattle technique [92,93] or insecticide-treated (odor-baited) targets [94]. Strategies for vector eradication include the sequential aerosol technique [95] involving the aerial spraying of pyrethroid formulations and the sterile insect technique [96] which unleashes irradiated males to sterilize wild females when they mate with them. A method described as paratransgenesis reduces or eliminates the ability of tsetse flies to transmit trypanosomes by compromising their microbiome through genetic modification. Genetically modified Sodalis introduced into the larval stages was found to be capable of efficiently colonizing the succeeding generations of offsprings, hence demonstrating that Sodalis can be employed as a vehicle to deliver foreign transgenes for the transformation of *Glossina morsitans morsitans* in paratransgenesis [97]. Other methods of control include ground spraying and bush clearing and game destruction. Odor-baited targets are more economical than aerial spraying and less harmful to the environment than insecticidal ground spraying, game destruction, or habitat clearance. However, it has only been used for extensive tsetse elimination in Zimbabwe and in the Zambian West Province because of the cost, the logistic requirements, and the need for government commitment [94]. Repellents can also be employed for vector control, for instance, the push-pull control method employs repellents to push the tsetse flies towards stationary visual attractants designed to kill the flies [98]. The repellent mix called waterbuck repellent compounds (WRC) and 4-methylguaiacol which were known *Mortisan* group tsetse fly repellents were tested for their effects on the *Palpalis* group and found to effectively repel *Glossina fuscipes fuscipes* [98]. Similarly, unrefined zebra skin odour, as well as a chemical mix with identical odor, was found to significantly repel *Glossina pallidipes* [99]. 

The African Union in 2000 adopted a policy to eradicate tsetse flies and trypanosomiasis from Africa following the successful and enduring elimination of *G. austeni* from Unguja Island in Zanzibar using the sterile insect technique, and the subsequent disappearance of the AAT disease [96]. This led to the setting up of the Pan African Tsetse and Trypanosomiasis Eradication Campaign (PATTEC) to coordinate programs aimed at tsetse fly and trypanosomiasis elimination across the continent of Africa [96]. However, the sterile insecticide technique was recommended for use after the tsetse population has initially been reduced by other methods, such as the combination of odor-baited targets and insecticide-treated cattle [94].

## 11. Challenges Facing HAT Elimination

As stated earlier, the notional global target of achieving a less than 2000 reported HAT cases by 2020 set by the WHO has already been met. The initial figures gathered by WHO showed reported cases in 2017 to be less than 1500 [34]. Despite this remarkable achievement, HAT remains a public health problem in some countries. This setback is due to a combination of factors converging to limit the progress towards elimination of HAT.

One of the most critical factors impeding the achievement of the WHO’s ambitious goal of elimination of HAT is identifying the level of drug resistance in the trypanosome populations in Africa. Even the anticipated introduction of the new treatment, fexinidazole, does not solve this problem as it has been found to be cross-resistant with the existing essential drug, nifurtimox [100]. Thus, an increased understanding of the incidence of resistant alleles (including for nifurtimox) in clinical isolates is vital to winning the end-game in finally erasing this scourge from sub-Saharan Africa. However, to date, very little is known about the spread of such resistance genes in the trypanosome species responsible for HAT in Africa.

Another of such factors is accessibility, which is still limited in some endemic areas [41], cases even go undetected or reported in some remote areas lacking access to medical facilities. This means that the actual figure may be higher than the <1500 cases reported by the WHO, and this is a real challenge. 

There is a growing body of evidence indicating that asymptomatic carriers persist in certain foci after medical surveys and cannot be diagnosed passively [101,102,103]. Similarly, both the skin [104,105] and adipose tissue [106] have been found recently to be the sites of multiplication of trypanosomes. This could explain the existence of serologically positive patients showing no trypanosomes in the blood. These seemingly aparasitaemic hosts harbored trypanosomes that were able to infect the tsetse flies [107]. Recent findings suggest that the infection could be from skin-dwelling trypanosomes taken up with the blood meal [105]. The problem here is that treatment is presently only provided for microscopy-positive subjects, leaving a percentage of the infected population of undetermined significance untreated.

The presence and emergence of higher-burden competing diseases is also an identified factor affecting the HAT elimination program. From a historical point of view, a successful elimination campaign can limit itself when national health authorities are struggling to defend the continued investment in the face of declining case numbers while at the same time confronting and/or prioritizing other, higher-burden diseases. A classic example of this is Guinea, where the Ebola outbreak of 2014 led to an interruption of the national HAT control program, and a corresponding increase in the HAT burden [108]. Political and social instability, inappropriate funding, and lack of ownership of the HAT problem by endemic countries are also part of the challenges facing the program to eliminate HAT.

The animal reservoirs of *T. b. gambiense* reported in West [109,110,111,112] and Central [113,114] Africa are additional factors that must be considered for a successful elimination program. It has been observed that patient-derived *T. b. gambiense* strains are capable of cycling between tsetse flies and various animal species without losing infectivity to humans [115]. Now, more attention is being given to cattle as a reservoir of trypanosomes, as several reports have underlined the significance of domestic and wild animals as important factors in the spreading of *T. b. rhodesiense* [114,116]. Studies have shown that domestic pigs could play an important role in the epidemiology of trypanosomiasis both in humans and animals [117]. Pigs are becoming important as a source of food and income earner for smallholder livestock farmers, especially in areas where AAT and both forms of HAT are endemic. Therefore, efforts directed towards bovine trypanosomiasis alone while ignoring other potential domestic animal reservoirs might be a contributory factor to the persistence of sleeping sickness in Africa. For instance, in a recent study of trypanosome species circulating in livestock in two HAT foci of Côte d’Ivoire, pigs and cattle were identified as reservoirs of *T. b. gambiense* [117].

A potential contradiction would arise when a very low number of new infections of HAT presents a reason for demotivation [103]. The potential complacency would be a cog in the wheel of progress towards this elimination drive. Donors’ demotivation when faced with the huge amount of money needed to be spent on surveillance and drug provision when few or no new patient(s) are being detected, technicians’ demotivation when thousands of people are being tested without finding new infections, the inhabitants being demotivated to participate in unending medical surveys with no new infection being found, are just a few. The event that hindered HAT elimination in the 1960s must be totally avoided to ensure the ultimate achievement of the set goal. Therefore, to achieve the WHO elimination goal, HAT control needs to be approached using the One Health strategy. This approach involves the formulation and execution of programs, policies, legislations, and research jointly by the agencies in charge of HAT and AAT elimination. Communication between these two groups of agencies is necessary to improve the public health outcome since trypanosomiasis can be zoonotic (https://www.who.int/features/qa/one-health/en/). Possible synergy at the interface of One Health can facilitate the simultaneous elimination of both AAT and HAT.

## 12. Insights for the Future 

Elimination of a disease can be achieved by ensuring that R_c_ (reproduction number of the disease in the presence of control) is below 1 for a significant amount of time [118]. For HAT, this will require the combined application of vector control methods, sensitive diagnostic tests, and trypanocidal drugs. *T. b. rhodesiense* is zoonotic, with wild and domestic animals as carriers. Since its infection in humans is usually acute, causing the infected subjects to try to obtain treatment soon after infection, the most practical method of combating rHAT would be the provision of excellent diagnostic tools and increasing the treatment options available at health centers in endemic areas. This approach would enhance early diagnosis and treatment of infection, and in combination with vector control, would ultimately break the transmission of this disease [110]. Conversely, humans are the reservoirs for *T. b. gambiense* [119], with HAT progressing for years if not treated [120]. Animal hosts of *T. b. gambiense* have also been identified [121,122], and this could be an important factor when considering the transmission of this disease. The three-way method suggested for gHAT control includes active case detection using mobile teams, passive case detection, treatment at hospitals and health centers, and vector control [110].

Results of recent simulations suggest that detection and treatment of gHAT infections using the currently registered trypanocides and diagnostic methods is ineffective and less likely to result in full elimination of gHAT by 2030 [67]. The introduction of fexinidazole and acoziborole is expected to remove side effects due to toxicity, reduce the cost of treatment and remove the requirement of hospitalization for treatment. If these new treatment regimens are supported with sensitive, point-of-care rapid diagnostic equipment, then case detection and treatment can easily be carried out in all locations: markets, schools, churches, offices, and so on, in endemic communities. With portable diagnostic equipment, the employment of motorbike surveillance teams for remote communities becomes feasible. Insecticide-treated targets can also be replaced with tiny targets that are very much smaller and cheaper than the odor-baited targets [67].

## 13. Conclusions

With the early achievement of the 2020 goal of a drastic reduction in the number of cases of HAT (thus reducing concerns of HAT as a public health problem), the goal of its sustainable elimination seems very promising. However, when one considers the resources presently available for this purpose, the goal of eliminating HAT seems very far-fetched. Diagnostic tools available needs to be improved to ensure prompt detection and treatment. New drugs for single and combination therapies which are non-toxic, affordable, easily administered, and with no cross-resistance to existing drugs need to be introduced. Drug resistance in African populations need to be monitored and characterized, and then active case-finding and surveillance need to be sustained until elimination is achieved. These would require a concerted investment of resources.

## Figures and Tables

**Table 1:** Natural products found to possess promising trypanocidal activities in the last decade.

S/No	Class of Compound	Name of Compound	Source	IC_50_ (µM)	Active against	Selectivity Index	Proposed Mechanism of Action	Ref
1	Piperidine Alkaloids	(+)-Spectaline	*Senna spectabilis* leaves	0.410±0.010	*Trypanosoma brucei rhodesiense*	135	Autophagic cell death resulting from mitochondrial damage due to interference of the sterol synthetic pathway in *Trypanosoma brucei rhodesiense.*	[74]
2	Piperidine Alkaloids	Iso-6-spectaline	*Senna spectabilis* leaves	0.710±0.010	*Trypanosoma brucei rhodesiense*	124	Autophagic cell death resulting from mitochondrial damage due to interference of the sterol synthetic pathway in *Trypanosoma brucei rhodesiense.*	[74]
3	Sesquiterpene	Isofuranodiene	*Smyrnium olusatium* parts (fruits, flowers, leaves and roots)	3.000±0.800	*Trypanosoma brucei brucei*	30	Apoptosis resulting from altered mitochondrial membrane permeability, inhibition of key enzymes involved in metabolism such as dihydrofolate reductase, reactivity with functional groups of biological molecules due to electron delocalization of the furan moiety.	[75]
4	Diterpene	16α-hydroxycleroda-3,13 (14)-Z-dien-15,16-olide	*Polyalthia longifolia* leaves	0.380±0.050 µg/mL	*Trypanosoma brucei brucei*	>526	Disruption of biological membranes in the parasite – leading to decreased fluidity, inhibition of membrane proteins hence signaling and transport.	[76]
5	Sesquiterpene lactone	Deoxyelephantopin	*Elephantopus scaber* leaves	0.070±0.015	*Trypanosoma brucei rhodesiense*	65	Inactivation of the immune system, due to bond formation with panthione, thereby exposing parasites to oxidative damage.	[77]
6	Sesquiterpene lactone	Vernodalin	*Vernonia cinerascens* leaves	0.160±0.040	*Trypanosoma brucei rhodesiense*	35	-	[78]
7	Sesquiterpene lactone	Vernolide	*Vernonia cinerascens* leaves	0.500±0.010	*Trypanosoma brucei rhodesiense*	13	-	[78]
8	Diterpene glycoside	Cupacinoside	*Cupania cinereal*	<10	*Trypanosoma brucei rhodesiense*		-	[79]
9	Pentacyclic triterpenoid	Taraxerol	*Cupania cinereal*	<10	*Trypanosoma brucei rhodesiense*	-	-	[79]
10	Triterpenic acid	Ursolic acid	*Keetia leucantha*	2.190±0.438	*Trypanosoma brucei brucei*	-	- Not stated. Similar compounds had previously been identified in other plants.	[80]
11	Triterpenic acid	Oleanolic acid	*Keetia leucantha*	6.131±1.095	*Trypanosoma brucei brucei*	-	- Not stated. Similar compounds had previously been identified in other plants.	[80]
12	Sesquiterpene lactone	Cynaropicrin	*Centaurea salmantica L.* aerial parts/*Cynara scolymus*	0.280±0.01	*Trypanosoma brucei rhodesiense*	8	Affected cell proliferation in bloodstream forms.	[81]
13	Monoterpene glycosides	(3S, 6R) cis-linalool 3,6 oxide, O-β-D-xylopyranosyl-(1″→6′)-β-D-glucopyranoside	*Vangueria edulis*	8.180 µg/mL	*Trypanosoma brucei brucei*	-	-	[82]
14	Monoterpene glycosides	Quercetin-7-o-ɑ-L-rhamnopyranoside	*Vangueria edulis*	9.020 µg/mL	*Trypanosoma brucei brucei*	-	-	[82]
15	Bromopyrrole alkaloids	Dibromopalau’amine	*Axinella verrucose*	0.460 μg/mL	*Trypanosoma brucei rhodesiense*	~10	Identified structural motifs in the compound associated with trypanocidal activity.	[83]
16	Bromopyrrole alkaloids	Longamide	*Agelas dispar*	4.936	*Trypanosoma brucei rhodesiense*	-	Identified structural motifs in the compound associated with trypanocidal activity.	[83]
17	Bromopyrrole alkaloids	Sceptrin	Four different *Agelas* sponges (*A. conifera*, *A. clathrodes*, *A. longissima*, *A. dispar*)	15.654	*Trypanosoma brucei rhodesiense*	-	Identified structural motifs in the compound associated with trypanocidal activity.	[83]
18	Bromopyrrole alkaloids	Spongiacidin B	*Axinella verrucose*	13.580 μg/mL	*Trypanosoma brucei rhodesiense*	-	Identified structural motifs in the compound associated with trypanocidal activity.	[83]
19	Sesquiterpene lactone	Xanthatin	*Xanthium strumarium* leaves	10.881	*Trypanosoma brucei brucei*	20	Weak irreversible inhibition of trypanothione reductase, inhibition of Prostaglandin E synthesis and 5-lipoxygenase activity thereby inducing apoptosis. Reduction in mitochondrial membrane potential.	[84]
20	Triterpenoid saponins	Heinsiagenin A 3-O-[α-L-rhamnopyranosyl-(1→2)-β-Dglucopyranosyl-(1→2)]-β-D-glucopyranoside	*Mussaenda luteola* aerial parts	8.800±0.640	*Trypanosoma brucei brucei*	>10	-	[82]
21	Triterpenoid saponins	Heinsiagenin A 3-O-[α-L-rhamnopyranosyl(1→2)-β-D-glucopyranosyl-(1→2)]-[β-D-glucopyranosyl-(1→4)]-β-D-glucopyranoside	*Mussaenda luteola* aerial parts	2.570±0.640	*Trypanosoma brucei brucei*	>10	-	[82]
22	Triterpenoid saponins	2α-hydroxyheinsiagenin A 3-O-[α-L-rhamnopyranosyl-(1→2)-β-D-glucopyranosyl-(1→2)]-β-Dglucopyranoside	*Mussaenda luteola* aerial parts	2.610±0.090	*Trypanosoma brucei brucei*	>10	-	[82]
23	Triterpenoid saponins	2α-hydroxyheinsiagenin A 3-O-[β-D-glucopyranosyl-(1→2)]-[β-Dglucopyranosyl-(1→4)]-β-D-glucopyranoside	*Mussaenda luteola* aerial parts	2.840±0.390	*Trypanosoma brucei brucei*	>10	-	[82]
24	Triterpenoid	Salvadione C	*Salvia hydrangea* aerial parts	4.330±0.240	*Trypanosoma brucei rhodesiense*	43	-	[85]
25	Triterpenoid	Perovskone B	*Salvia hydrangea* aerial parts	15.920±0.720	*Trypanosoma brucei rhodesiense*	1	-	[85]
26	Isoflavonoids	Abruquinones K	*Abrus precatorius*	0.110±0.053	*Trypanosoma brucei rhodesiense*	509	-	[86]
27	Isoflavonoids	Abruquinones L	*Abrus precatorius*	0.020±0.003	*Trypanosoma brucei rhodesiense*	374	-	[86]
28	Isoflavonoids	Abruquinones A	*Abrus precatorius*	0.020±0.003	*Trypanosoma brucei rhodesiense*	1379	-	[86]
29	Isoflavonoids	Abruquinones D	*Abrus precatorius*	0.010±0.001	*Trypanosoma brucei rhodesiense*	668	-	[86]
30	Lanosane triterpenoids	Hexatenuins A	*Hexagonia tenuis* (Fungi)	0.570 μg/mL	*Trypanosoma brucei brucei*	-	-	[87]
31	Lanosane triterpenoids	Hexatenuins B	*Hexagonia tenuis* (Fungi)	8.600 μg/mL	*Trypanosoma brucei brucei*	-	-	[87]
32	Lanosane triterpenoids	Hexatenuins C	*Hexagonia tenuis* (Fungi)	5.620 μg/mL	*Trypanosoma brucei brucei*	-	-	[87]
